# Pathogenicity and Genomic Characterization of *Vibrio parahaemolyticus* VSP1: A Pathogen Linked to Enteritis Outbreak in Shrimp (*Penaeus vannamei*)

**DOI:** 10.3390/pathogens14111188

**Published:** 2025-11-20

**Authors:** Jing Wang, Fengguang Shen, Meng Tian, Fanqi Zeng, Lei Huang, Jiayun Yao, Can Zong, Jiong Chen, Demin Zhang, Haipeng Guo

**Affiliations:** 1 State Key Laboratory for Quality and Safety of Agro-Products, School of Marine Sciences, Ningbo University, Ningbo 315211, China; 2201130081@nbu.edu.cn (J.W.); shenfengguang@163.com (F.S.); 226004438@nbu.edu.cn (M.T.); 236004639@nbu.edu.cn (F.Z.); 246003089@nbu.edu.cn (C.Z.); chenjiong@nbu.edu.cn (J.C.); zhangdemin@nbu.edu.cn (D.Z.); 2 Key Laboratory of Aquacultural Biotechnology, Ministry of Education, Ningbo University, Ningbo 315211, China; 3 Zhejiang Institute of Freshwater Fisheries, Huzhou 313001, China; huanglein@sina.com (L.H.); yaojiayun@126.com (J.Y.)

**Keywords:** *Penaeus vannamei*, enteritis disease, *Vibrio parahaemolyticus*, genomic analysis, virulence factors

## Abstract

Enteritis is a common and recurrent disease in shrimp aquaculture, causing significant economic losses and management challenges. However, its specific causative pathogen remains unclear. Here, a pathogen strain, *Vibrio parahaemolyticus* VSP1, was directly isolated from shrimp with enteritis, and its pathogenicity and genomic characteristics were analyzed. Diseased shrimp exhibited lethargy, empty gut, hepatopancreatic atrophy, and severe intestinal damage. The gut bacterial community of diseased shrimp differed significantly from healthy shrimp (PERMANOVA, *p* < 0.05), with a 129% increase in *Vibrio* relative abundance. Nine *Vibrio* operational taxonomic units (OTUs) were enriched in diseased shrimp, and the dominant OTU1 shared 100% 16S rRNA identity with VSP1. VSP1 grew rapidly, utilized diverse carbon sources, and induced enteritis symptoms in over 90% of challenged shrimp. Genome analysis revealed 98.34% average nucleotide identity with *V. parahaemolyticus* ATCC 17802 and identified 156 putative virulence-related genes, mainly related to adherence, motility, and secretion systems. Unlike the strain ATCC 17802, VSP1 lacks thermostable direct hemolysin (TDH) and type III secretion system 2 (T3SS2), but contains alternative virulence factors such as *Yersinia*-like type IV pili and lipooligosaccharides, suggesting a distinct virulence strategy. This study identifies the pathogen responsible for shrimp enteritis and provides a foundation for targeted control strategies in aquaculture.

## 1. Introduction

*Penaeus vannamei*, also known as the Pacific white shrimp, is the world’s most widely farmed shrimp, playing a key role in global aquaculture due to its fast growth and adaptability [1]. However, as intensive shrimp farming expands, diseases have emerged as a significant threat to both the health and sustainability of shrimp aquaculture [2,3]. In recent years, shrimp enteritis has emerged as a common and recurrent disease in intensive shrimp aquaculture systems, typically presenting with chronic intestinal inflammation, impaired digestion, and stunted growth [4]. This disease often extends production cycles by 15 to 30 days, causing significant economic losses and persistent management difficulties for farmers [5,6,7].

Identifying the etiology is crucial for disease treatment of shrimp enteritis. Certain studies have been suggested that shrimp enteritis might be caused by bacterial pathogens, including novel *Vibrio harveyi*, *Streptococcus iniae* and novel *Vibrio* spp. etc. [8,9,10]. However, the specific pathogen that caused the enteritis of shrimp remains unclear. Koch’s postulates have long been considered the gold standard for identifying the causative agents of diseases based on the isolation of various potential pathogens [11,12], but the large-scale and non-directional isolation and cultivation of pathogenic strains is time-consuming and labor-intensive. High-throughput sequencing has been used to identify and characterize pathogens in various clinical samples [13,14], and provides more opportunities to guide the isolation and cultivation of potential pathogenic strains. Therefore, integrating high-throughput sequencing with Koch’s postulates might help to identify the key pathogen associated with shrimp enteritis rapidly [15].

Bacterial pathogenicity is closely associated with a diverse array of virulence determinants, including hemolysins (e.g., *tdh*, *trh*) [16], type III/VI secretory systems (T3SS/T6SS), flagellar motility proteins, and prophage-associated toxins [17], as well as polysaccharide biosynthesis clusters [18,19]. These factors are mainly involved in host colonization, immune evasion, and tissue damage, leading to various pathological characteristics, such as hepatopancreatic necrosis and intestinal epithelial sloughing [20]. Genes related to these virulence determinants are responsible for bacterial pathogenicity. Whole genome sequencing (WGS) has emerged as a powerful tool for characterizing these virulence genes, resistance elements, and metabolic traits at high resolution, thereby providing critical insights into the genetic basis of pathogenicity [21,22,23,24].

Here, a potential pathogen that causes the shrimp enteritis was directly isolated based on the results of high-throughput sequencing analysis from the diseased shrimp. The pathogen was identified as the *Vibrio parahaemolyticus* VSP1, and its pathogenicity was verified based on Koch’s postulates. Then, the whole genome sequencing and comparative genomic analysis were employed to investigate the genetic basis associated with its pathogenicity. 

## 2. Materials and Methods

### 2.1. Sample Collection and Experiment Shrimp

In May 2023, a massive enteritis outbreak occurred in the biggest shrimp farm in Ninghai County, Zhejiang Province, which caused more than 60% infection of shrimp in over 100 high-level ponds. The disease was primarily characterized by empty and reddened guts, along with diminished shrimp vitality, but it did not result in large-scale mortality. Shrimp samples were collected from five aquaculture ponds experiencing disease outbreaks. Twenty diseased shrimp, exhibiting typical signs of enteritis such as reduced vitality, empty stomachs and guts, were randomly selected from these ponds. In contrast, healthy shrimp were collected from disease-free ponds, showing active behavior and well-fed guts. All shrimp were surface-sterilized with 75% ethanol, and dissected aseptically to obtain the gut samples. The samples were then divided into two portions: one part was fixed in 4% paraformaldehyde for histopathological analysis, and the other part was immediately frozen in liquid nitrogen, and stored at −80 °C for gut bacterial community analysis. In addition, some gut samples from diseased shrimp were used for the isolation of pathogenic bacteria.

### 2.2. Tissue Sectioning and H&E Staining

Gut tissues from shrimp were fixed in 4% paraformaldehyde at 4 °C for 24 h, then transferred to 70% ethanol for storage. The fixed samples were dehydrated through a graded series of ethanol and xylene, and embedded in paraffin [25]. Thin sections of 5 μm were prepared using a Leica RM2135 rotary microtome (Leica Microsystems GmbH, Wetzlar, Germany). The sections were stained with hematoxylin and eosin (H&E) to highlight tissue structures and subsequently examined under a Zeiss LSM780 laser scanning confocal microscope (Carl Zeiss SAS, Jena, Germany).

### 2.3. DNA Extraction and Full-Length 16S rRNA Gene Sequencing

Bacterial DNA was extracted from shrimp gut samples using the QIAamp^®^ DNA Stool Mini Kit (Qiagen, Hilden, Germany) according the manufacturer’s instructions. DNA concentration and purity were determined using a NanoDrop 2000 spectrophotometer (Thermo Fisher Scientific, Waltham, MA, USA). The full-length 16S rRNA genes were amplified by polymerase chain reaction (PCR) with primers 27F (5′-AGAGTTTGATCMTGGCTCAG-3′) and 1492R (5′-TACGGYTACCTTGTTAYGACTT-3′). The PCR products were purified using a PCR Fragment Purification Kit (TaKaRa Biotechnology, Kusatsu, Japan), and then sequenced on the PacBio RS II platform (Pacific Biosciences of California, Menlo Park, CA, USA) at Novogene Co., Ltd. (Beijing, China).

### 2.4. Sequencing Data Analysis

Raw PacBio sequencing data were demultiplexed using Lima (with same and peek-guess flags) based on barcode sequences. Circular consensus sequencing (CCS) reads were then generated using SMRT Link (v7.0) with parameters set to CCS ≥ 3, and a minimal predicted accuracy of 99% [26]. Sequences shorter than 1340 bp or longer than 1640 bp were removed. Primer sequences were trimmed using Cutadapt (v4.2), and reads containing homopolymers longer than eight bases were filtered out. The resulting high-quality sequences were designated as clean reads. Clean reads were clustered into operational taxonomic units (OTUs) at 99% similarity using CD-HIT (v4.8.1) [27]. Taxonomic annotation was conducted using the UCLUST method [28], applying similarity thresholds of 0.8 and 0.9. The annotation was carried out with the SILVA SSU rRNA database (v138.1) [29], and the GTDB 16S rRNA database (v1.0) [30].

Shannon, Simpson, Chao1, and Pielou indices were calculated using the R package ‘vegan’ to evaluate alpha diversity of the shrimp gut bacterial community [31]. Principal coordinate analysis (PCoA) based on Bray-Curtis dissimilarity was used to visualize community structure. Bacterial community differences between healthy and diseased shrimp were assessed using permutation multivariate analysis of variance (PERMANOVA) with the ‘vegan’ package in R [32]. The discriminatory OTUs were analyzed with the standards of (|log_2_Fold Change|) > 2.0 and *p*-value from FDA < 0.05 using the ‘DESeq2’ package in R.

### 2.5. Isolation and Identification of Potential Pathogens

According to the discriminatory OTUs, assigned as *Vibrio* were the main enriched taxa in the diseased shrimp. Therefore, the bacteria belonging to *Vibrio* were directly isolated from the diseased shrimp using thiosulfate citrate bile salts sucrose (TCBS) agar. Briefly, gut contents of diseased shrimp were collected under sterile conditions, and homogenized to prepare the suspension by adding 1 mL of sterile phosphate-buffered saline (PBS). Then, 100 µL of the suspension was taken for serial dilution, and the dilutions were plated onto TCBS agar plates, which were then incubated at 30 °C for 24 h. Two distinct bacterial strains, VSP1 and VSP2, were isolated based on colony morphology. The isolates were cultured in marine 2216E medium at 30 °C with shaking at 200 rpm for 24 h. Genomic DNA was extracted using a 10% Chelex solution, and the full-length 16S rRNA gene was amplified by PCR with primers 27F (5′-AGAGTTTGATCMTGGCTCAG-3′) and 1492R (5′-TACGGYTACCTTGTTAYGACTT-3′). The PCR products were sequenced using an Applied Biosystems 3730 DNA sequencer (Applied Biosystems, Foster City, CA, USA), and the Sanger sequencing platform. The 16S rRNA gene sequences of VSP1 and VSP2 were aligned as the subject sequence to match the discriminatory OTU sequences (Query sequence) by the basic local alignment search tool searches on NCBI (https://blast.ncbi.nlm.nih.gov/Blast.cgi, accessed on 1 May 2024). The phylogenetic tree was constructed using the neighbor-joining (NJ) method with 2000 bootstrap replications in MEGA (v10.0). Evolutionary distances were calculated using the *p*-distance model with pairwise deletion of ambiguous positions.

### 2.6. Virulence Test with the Isolates

To evaluate the pathogenicity of the isolated strains, healthy shrimp (approximately 5 g per individual) were obtained from a farm in Xiangshan, Ningbo, China, and used for virulence test. The experiment was conducted in a 70-L aerated tank containing 50 L of water and 20 shrimp. Prior to the experiment, the isolated strains were inoculated into marine 2216E medium and incubated at 30 °C with shaking at 200 rpm overnight. The bacterial cultures were then centrifuged at 4000 rpm for 10 min. After discarding the supernatant, the bacterial pellets were resuspended in sterile seawater to an optical density at 600 nm (OD_600_) of approximately 1.0, corresponding to ~1 × 10^9^ colony-forming units (CFU) mL^−1^. The challenge test was conducted according to our previous studies [33,34]. Briefly, shrimp in each tank were initially exposed to 5 L of a VSP1 or VSP2 suspension (~1 × 10^8^ CFU mL^−1^) for 15 min. Then 45 L of sterilized seawater was added into the tank, resulting in a final pathogen concentration of ~1 × 10^7^ CFU mL^−1^. The control group was maintained in 50 L of sterilized seawater without the addition of pathogens. Each treatment was performed in six replicates. The test was conducted for 24 h, during which shrimp were fed every 6 h. Shrimp activity and behavior was observed, and the numbers of surviving and infected shrimp were recorded to calculate the survival and infection rates. Shrimp gut samples were collected to isolate pathogenic bacteria, as above described.

### 2.7. Growth Curve and Carbon Source Utilization of the Isolates

The isolated strains were inoculated into marine 2216E medium, and pre-incubated at 30 °C, 200 rpm until the exponential phase. A 2% (*v*/*v*) inoculum was then transferred to fresh medium and cultured for 24 h under the same conditions. The OD_600_ values was measured every 2 h using a microplate reader. Three biological replicates were performed to ensure accuracy.

The carbon source utilization of the isolated strains was assessed using Biolog GEN III microplate (Biolog, Hayward, CA, USA) following the manufacturer’s instructions. Briefly, a bacterial suspension was prepared by adjusting the OD_600_ value to 0.01 with IF A solution. The suspension was inoculated into the microplate wells containing 71 carbon sources. Each well added 100 µL of the bacterial suspension, and the plate was sealed with adhesive film to prevent evaporation. Plates were incubated at 30 °C, 200 rpm for 24 h. After incubation, absorbance at 590 nm was measured to assess metabolic activity. The 590 nm reading reflects the color change resulting from the reduction of tetrazolium redox dye, which occurs during carbon oxidation, thus, it serves as a proxy for carbon utilization efficiency. To correct for background signal, values were normalized by subtracting the absorbance of the negative control well (A1). A carbon source was considered utilized when the corrected absorbance exceeded 0.1 [35].

### 2.8. Genomic Analysis of the Isolates

The genomic DNA of isolated strains was extracted using the TIANamp Bacteria DNA Kit (Tiangen Biotech (Beijing) Co., Ltd., Beijing, China) following the manufacturer’s instructions. The purity and integrity of DNA were assessed using agarose gel electrophoresis, and the concentration was quantified with a Qubit fluorometer (Thermo Fisher Scientific, Waltham, MA, USA). Qualified DNA samples were randomly fragmented into approximately 350 bp segments using a Covaris ultrasonicator (Covaris, Woburn, MA, USA). The fragmented DNA was then subjected to end repair, A-tailing, adaptor ligation, purification, and PCR amplification to construct the sequencing library using the NEBNext^®^ Ultra™ DNA Library Prep Kit for Illumina (New England Biolabs, Ipswich, MA, USA). Whole-genome sequencing as performed on the Illumina NovaSeq PE150 platform (Illumina, San Diego, CA, USA) at Beijing Novogene Bioinformatics Technology Co., Ltd. The raw data were initially processed to generate clean reads by filtering out adapter sequences and low-quality reads. Subsequently, all high-quality paired-end reads were assembled into multiple scaffolds using SOAP denovo (v2.04) [36]. Genome assembly quality, including completeness and contamination, as well as genome size and GC content, was assessed using CheckM2 (v1.0.2). Gene prediction and functional annotation were performed using Bakta (v1.9.4). Genes annotated as “hypothetical proteins” or “DUFXXX-like proteins” were excluded when counting the number of functionally annotated genes. The prephages in the genome were predicted by phiSpy (v2.3). In addition, virulence-related genes were identified using the Virulence Factors Database (https://www.mgc.ac.cn/VFs/, accessed on 1 November 2025). Resistance-related genes were annotated using the Comprehensive Antibiotic Resistance Database (CARD, v4.0.1) via the online RGI tool (https://card.mcmaster.ca/analyze/rgi, accessed on 1 October 2025) with stringent criteria: only Perfect and Strict hits were considered, and partial gene predictions were excluded. To identify the species of VSP1 and VSP2, the genomes of the 15 *Vibrio* type strains were downloaded from NCBI (Table S1), and the Average Nucleotide Identity (ANI) was calculated using FastANI with default parameters. Strains with an ANI ≥ 95% were considered to belong to the same species [37]. All statistical analyses and data visualization were conducted using R software (v4.3.3).

### 2.9. Antimicrobial Susceptibility Testing

Antimicrobial susceptibility testing was performed using the Kirby–Bauer disk diffusion method on Mueller–Hinton agar [38]. Briefly, bacterial suspensions standardized to a 0.5 McFarland turbidity were plated onto MH agar plates, followed by the placement of antibiotic discs. After incubation at 37 °C for 16–18 h, the diameters of inhibition zones were measured and interpreted as resistant (R), intermediate (I), or susceptible (S) according to the Clinical and Laboratory Standards Institute (CLSI) guidelines [39]. A total of 12 antibiotics representing seven major classes were tested: β-lactams—ampicillin (AMP), piperacillin (PIP), cefotaxime (CTX), cefoxitin (FOX), cefepime (FEP), imipenem (IPM), ceftazidime (CAZ); fluoroquinolone—levofloxacin (LEV); quinolone—ciprofloxacin (CIP); tetracycline—tetracycline (TE); aminoglycoside—gentamicin (CEN); and phenicol—chloramphenicol (CMP).

## 3. Results

### 3.1. Clinical Signs and Histopathological Characterization of Diseased Shrimp

**Figure 1:**
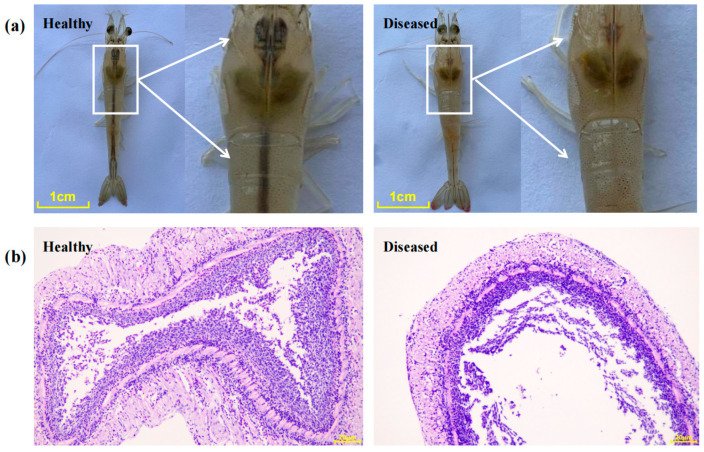
Clinical and histopathological examination of shrimp with enteritis. (**a**) Phenotypic characteristics; (**b**) Histological sections of the gut. Bar = 1 cm in (**a**), and 20 μm in (**b**).

Clinical observations revealed that shrimp from the disease-free pond exhibited good vitality, normal feeding behavior, a dark-colored stomach, an intestine filled with feed, and a hepatopancreas of normal size with a uniform brown coloration. In contrast, shrimp from the diseased pond exhibited clear signs of lethargy and had empty gastrointestinal tracts. The hepatopancreas was slightly atrophied and appeared dark brown (Figure 1a). Histological examination demonstrated that the gut structure of healthy shrimp was intact with well-organized layers, including a clearly defined mucosa, submucosa, and muscularis externa. The epithelial cells were tightly arranged with no signs of detachment. In contrast, the gut of diseased shrimp exhibited significant pathological changes, including thinning of the intestinal wall, notably reduced muscularis externa and submucosa layers, disorganized epithelial cells, and increased epithelial cell detachment (Figure 1b).

### 3.2. Screening of the Potential Pathogens Based on the Gut Bacterial Community Analysis

**Figure 2:**
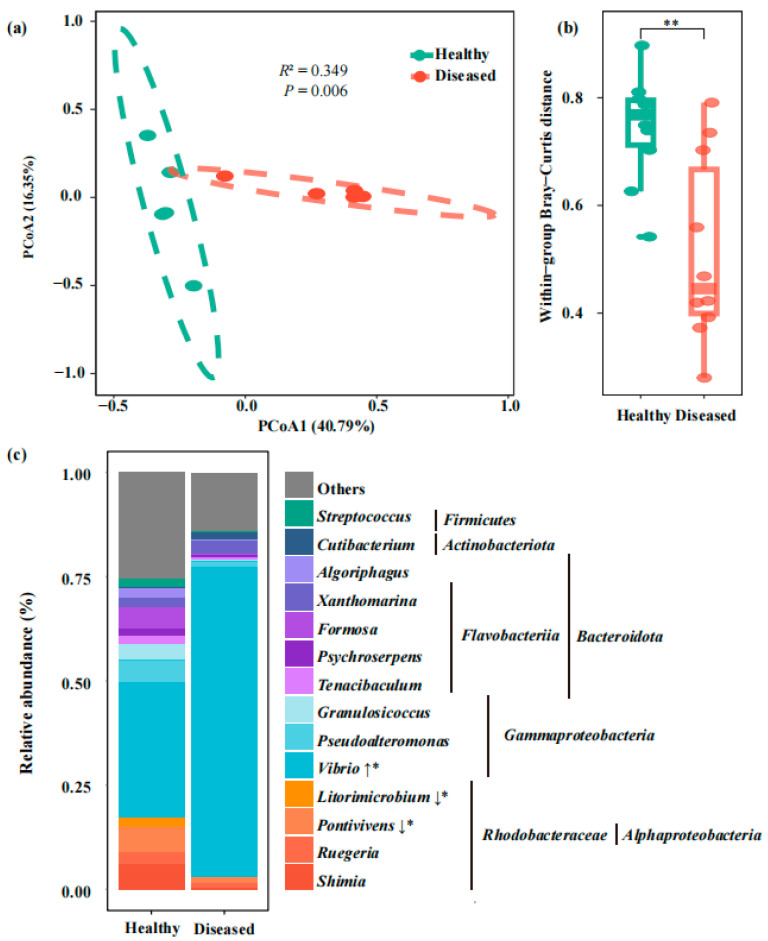
Gut bacterial community differences between healthy and diseased shrimp. (**a**) PCoA based on Bray-Curtis dissimilarity. (**b**) Within-group distance of gut bacterial communities (** indicates *p* < 0.01, Wilcoxon rank-sum test). (**c**) Relative abundance of dominant genera. Genera significantly enriched or depleted compared to healthy shrimp are marked with ‘↑*’ and ‘↓*’, respectively (*p* < 0.05, Wilcoxon rank-sum test). Each group includes five replicates (*n* = 5).

To explore whether shrimp enteritis is associated with gut bacterial dysbiosis, the gut bacterial communities of healthy and diseased shrimp were analyzed. Although no significant differences were observed in α-diversity indices between diseased and healthy shrimp (*p* > 0.05; Figure S1), the gut bacterial community structures were significantly different based on PCoA (PERMANOVA, *p* < 0.05), with more than 57% variances explained by the PCoA1 and PCoA2 together (Figure 2a). Furthermore, the gut bacterial communities of diseased shrimp showed a more convergent structure compared to healthy shrimp, as reflected by the lower within-group distances (Figure 2b). For the bacterial community composition, the relative abundance of *Vibrio* was significantly increased by 129% in diseased shrimp, while the relative abundances of taxa from *Rhodobacteraceae* were markedly reduced in diseased shrimp, compared to that in healthy shrimp (Figure 2c). These results indicated that the enrichment of *Vibrio* in shrimp might be closely associated with the shrimp enteritis.

**Figure 3:**
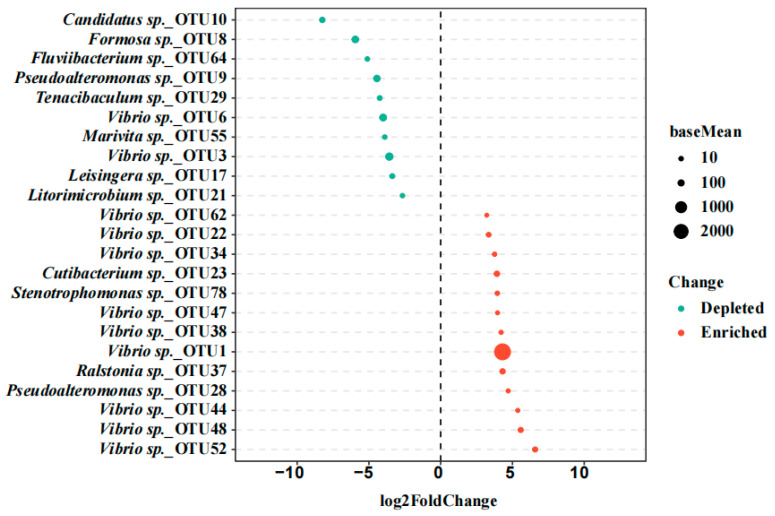
Fold changes of significantly depleted and enriched OTUs in diseased shrimp, compared to that in healthy shrimp (*P*_FDA_ *p*-value from FDA < 0.05).

To further identify discriminatory taxa between diseased and healthy shrimp, DESeq2 analysis based on OTU counts were conducted (Figure 3). A total of 23 OTUs were identified as significantly discriminatory taxa, including 10 depleted OTUs, and 13 enriched OTUs in diseased shrimp. Most notably, nine of the enriched OTUs were assigned to *Vibrio*, especially OTU1, which exhibited the highest relative abundance among all OTUs.

### 3.3. Targeted Isolation and Virulence Test of the Pathogen

**Figure 4:**
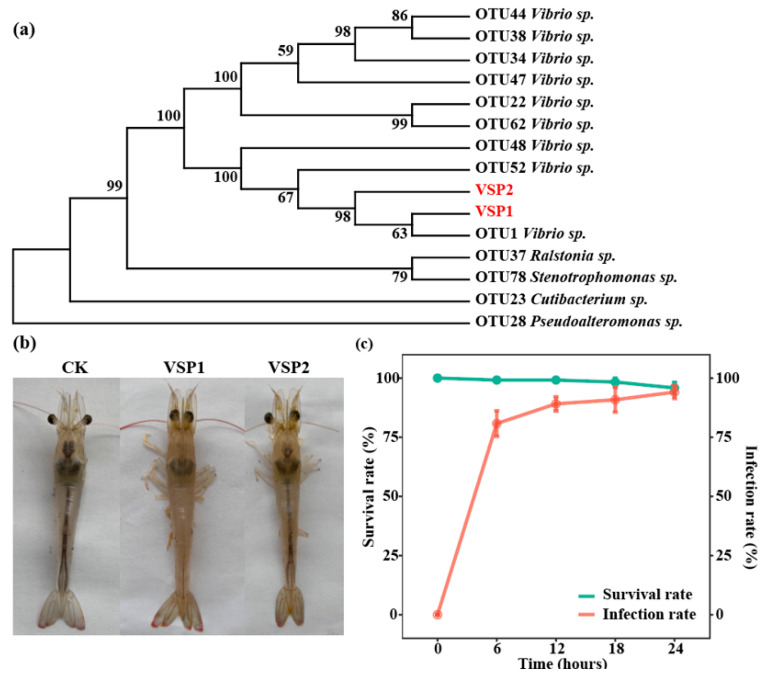
Isolation, identification, and virulence test of *Vibrio* strains. (**a**) Neighbor-joining phylogenetic tree showing the relationship between isolated strains VSP1 and VSP2, and OTUs enriched in diseased shrimp. (**b**) Clinical signs of shrimp after infection with VSP1 and VSP2. (**c**) Survival and infection rate of shrimp exposed to VSP1. The solid line indicates survival rate, and the dashed line represents infection rate (*n* = 6).

Given the significant enrichment of *Vibrio* spp. in diseased shrimp and their known pathogenicity in marine farmed animals, *Vibrio* strains were selectively isolated from the diseased shrimp gut using TCBS agar. Two strains, which showed different morphologies, were isolated and named as VSP1 and VSP2, respectively. The 16S rRNA gene of both isolates was sequenced, and compared with the sequences of enriched OTUs. The 16S rRNA gene sequence of VSP1 was 100% similar to the full-length sequence of OTU1 (Figure 4a), the most significantly enriched taxon in diseased shrimp, while VSP2 did not match any discriminatory OTUs.

To evaluate the pathogenic potential of the isolated strains VSP1 and VSP2, virulence test was conducted using healthy shrimp. After exposure to VSP1, more than 80% of shrimp were infected within 6 h, and exhibited the same clinical signs of enteritis (Figure 4b), and the ratios of infected shrimp were gradually increasing to 90% and 94% at 12 h and 24 h, respectively (Figure 4c). Despite the rapid onset of clinical signs, 96% of shrimp remained alive at 24 h post-infection, which was similar with the phenomenon found in the farming ponds. In contrast, no clinical symptom of disease or mortality were observed in shrimp challenged with VSP2 (Figure 4b). To further confirm that the VSP1 was the pathogen of shrimp enteritis, the VSP1 was successfully re-isolated from the infected shrimp, thereby fulfilling the Koch’s postulates, and confirming it as the causative agent of shrimp enteritis.

### 3.4. Biological Characteristics of the Isolated Strains

**Figure 5:**
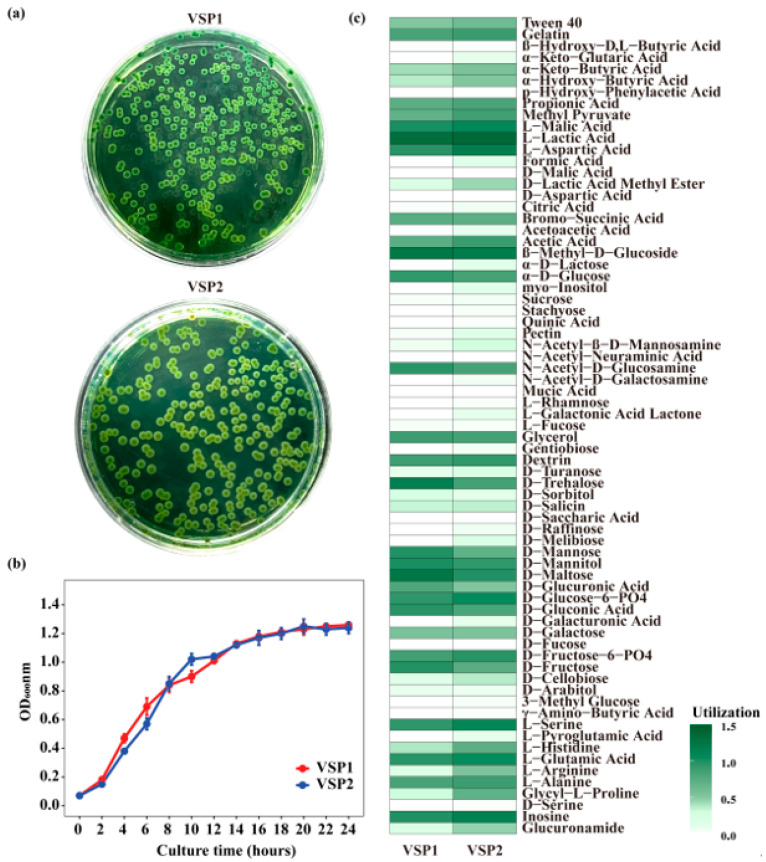
Morphology, growth curve, and carbon source utilization of VSP1 and VSP2. (**a**) Colony morphology on TCBS agar, (**b**) growth curves in marine 2216E medium, (**c**) carbon source utilization profiles based on Biolog GEN III MicroPlate.

To understand the biological characteristics of the isolated strains, their morphology, growth curves, and carbon source utilization profiles were analyzed. VSP1 formed a smooth, circular, green color colony on TCBS agar, while VSP2 exhibited similar colony morphology but with slightly yellowish edges (Figure 5a). The growth curve demonstrated that VSP1 grew rapidly, and reached the stationary phase at approximately 18–20 h, with an OD_600_ value of 1.2, while VSP2 showed a comparable growth trend but with a slightly slower increase during the early exponential phase (Figure 5b). Both VSP1 and VSP2 were able to metabolize a wide variety of carbon sources, including sugars (e.g., α-D-glucose, D-mannose, D-maltose, D-fructose), sugar alcohols (e.g., D-mannitol, D-sorbitol), organic acids (e.g., propionic acid, acetic acid, L-malic acid, L-lactic acid), and several amino acids (e.g., L-alanine, L-arginine, L-glutamic acid) (Figure 5c). In addition, both strains utilized polymers such as Tween 40 and gelatin. VSP2 also exhibited weak positive reactions to a few additional carbon sources, including D-turanose, D-melibiose, N-acetyl-β-D-mannosamine, myo-inositol, pectin, and formic acid.

### 3.5. The Genome Characteristics of the Isolated Strains

To investigate the genomic basis of virulence differences between the isolated strains, whole-genome sequencing was performed for both VSP1 and VSP2 using the Illumina NovaSeq PE150 platform. The assembled genomes of VSP1 and VSP2 were 5,366,644 bp and 5,603,652 bp in size, respectively, with a GC content of 45.0% in both strains (Table 1). Genome quality assessment using CheckM2 revealed 100% completeness and 0.08% contamination for VSP1, and 100% completeness and 2.12% contamination for VSP2. Gene prediction and functional annotation using Bakta identified 4829 and 5037 coding sequences (CDSs) in VSP1 and VSP2, respectively, among which 4276 and 4471 genes were functionally annotated (Table 1). In addition, VSP1 harbored 25 prophage regions, whereas VSP2 contained 17.

**Figure 6:**
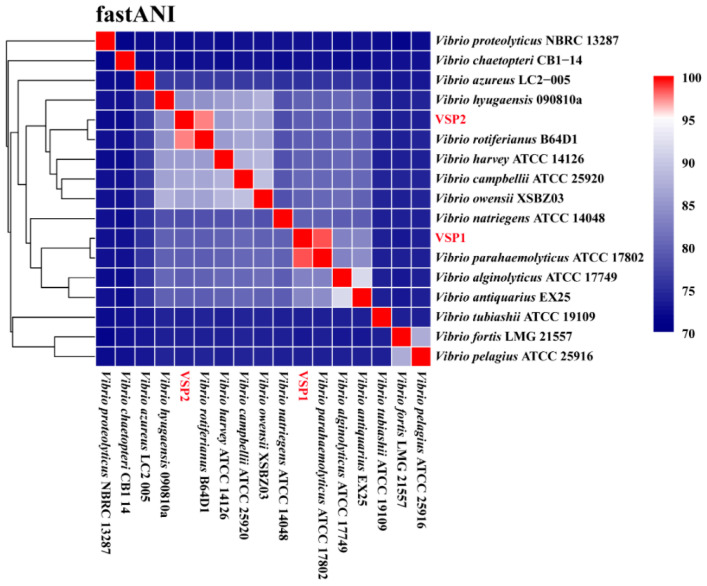
ANI analysis between VSP1, VSP2, and 15 type strains of *Vibrio* spp. The ANI values are represented by a color gradient, with red indicating high sequence similarity and blue indicating low similarity.

To clarify the taxonomic positions of the two isolates, ANI analysis was conducted between VSP1, VSP2, and 15 type strains of *Vibrio* spp. (Figure 6). The results demonstrated that VSP1 exhibited the highest ANI value (98.34%) with *Vibrio parahaemolyticus* ATCC 17802, whereas VSP2 showed the highest ANI value (97.44%) with *Vibrio rotiferianus* B64D1 (Figure 6), confirming that VSP1 and VSP2 could be definitively classified as *V. parahaemolyticus* and *V. rotiferianus*, respectively.

### 3.6. Virulence and Antibiotic Resistance Genes of the Isolated Strains

To elucidate the genomic determinants underlying the virulence of the isolated strains, the predicted genes were annotated using the VFDB database. A total of 156 putative virulence-associated genes were identified in the VSP1 genome, and these genes were assigned to 11 categories, mainly including adherence (20 genes), antiphagocytosis (15), motility (57), iron acquisition (6), secretion system (50), quorum sensing factor (2), toxin (1), etc. (Table 2). Among them, the genes related to adherence mainly included MSHA type IV pilus (15), type IV pilus (4), and *Yersinia* type IV pili (1), and the gene associated with motility was mediated by the flagellar (57), while the genes involved in secretion system mainly consisted of EPS type II secretion system (12), T3SS1 (34), T3SS1 secreted effectors (4). By contrast, 153 putative virulence-associated genes were identified in VSP2, which exhibited different virulence gene profiles with VSP1 (Table 2 and Table S2). For examples, VSP2 completely lacked the T3SS1 and its secreted effectors, as well as several adherence factors present in VSP1, such as the type IV pilus. Meanwhile, VSP2 possessed virulence-related genes absent in VSP1, including acinetobactin iron acquisition system and type VI secretion system (T6SS).

According to the ANI analysis, strain VSP1 showed the highest similarity to *V. parahaemolyticus* ATCC 17802. To further assess its virulence potential within this species, virulence-associated genes of VSP1 were compared with those of the highly pathogenic reference strain ATCC 17802. The results showed that VSP1 shared a similar virulence gene profile with strain ATCC 17802 but lacked several classical virulence determinants. Notably, VSP1 was missing the type III secretion system 2 (T3SS2) and its associated effectors, as well as the thermostable direct hemolysin (TDH) and alpha-hemolysin genes, which are typically linked to high virulence in clinical strains. In contrast, VSP1 possessed unique genes related to type IV pili (*Yersinia*), lipooligosaccharide (LOS), and an additional capsule gene, suggesting it may rely on alternative virulence mechanisms distinct from typical pathogenic *V. parahaemolyticus* strains (Table 2 and Table S2).

To assess the antibiotic resistance potential of the VSP1 and VSP2 strains, antibiotic resistance genes (ARGs) were annotated using the CARD database. The results showed that the genome of two strains all harbored six ARGs, and the ARGs mainly included *CARB-18*, *CRP*, *TxR*, *parE*, *adeF*, and *vanT* in VSP1 genome, and *CRP*, *parE*, *adeF*, *vanT*, *tet(B),* and *PBP3* in VSP2 genome (Table 3). These ARGs were mainly associated with resistance to β-lactams, macrolides, fluoroquinolones, tetracyclines, and glycopeptides. To validate the genomic predictions, antibiotic susceptibility testing was performed using the Kirby–Bauer disk diffusion method. The results were generally consistent with the genomic analysis. Both VSP1 and VSP2 exhibited resistance to multiple antibiotics, such as ampicillin, levofloxacin, and tetracycline, while remaining susceptible to cefoxitin and chloramphenicol (Table 4).

## 4. Discussion

### 4.1. V. parahaemolyticus VSP1 Is the Pathogenic Bacterium Causing the Enteritis in Shrimp

The rapid development of intensive aquaculture, particularly in high-density farming environments, has led to frequent outbreaks of enteritis in shrimp, causing significant economic losses in global aquaculture industries [40,41]. Previous studies have indicated that shrimp suffering from bacterial enteritis are typically characterized by reduced appetite, lethargy, slow growth, and erratic swimming. The hepatopancreas is frequently atrophied, pale to white in color, and may contain melanized lesions. The intestine usually appears empty or watery, with segmental discontinuities [42,43]. Histopathological characteristics of enteritis mainly include intestinal villus atrophy, submucosal inflammatory infiltration, epithelial desquamation or mucosal erosion, and in some cases concurrent hepatopancreatic necrosis [44,45,46]. In this study, shrimp with enteritis exhibited typical clinical and histological features—such as lethargic motility, empty stomach and gut—consistent with previous reports [47,48]. However, clinical treatments for shrimp enteritis have generally been ineffective, largely due to the unclear identification of the causative pathogens.

Accurate identification of the causative pathogen is essential for the effective treatment of shrimp enteritis. Traditionally, pathogen screening relies on isolation followed by experimental validation, which is often time-consuming and inefficient [11]. Recent advances in high-throughput sequencing technologies now allow for targeted screening of potential pathogens based on shifts in the gut bacterial community, providing a more rapid and reliable means of pathogen discovery [15]. In this study, analysis of the gut bacterial community revealed clear differences between healthy and diseased shrimp. The diseased group showed a marked enrichment of *Vibrio* spp., consistent with previous findings that overgrowth of *Vibrio* can drive the shift from a healthy to a diseased state [13]. Therefore, in this study, *Vibrio* strains were selectively isolated from diseased shrimp, and their pathogenicity was assessed through virulence testing. The results demonstrated that strain VSP1, isolated from diseased shrimp, could rapidly infect healthy shrimp and induce pathological signs consistent with enteritis. VSP1 was subsequently re-isolated from the experimentally infected shrimp. Thus, based on gut bacterial community analysis and in accordance with Koch’s postulates [12], VSP1 was confirmed as the pathogenic bacterium causing shrimp enteritis.

### 4.2. VSP1 Has Multiple Virulence Factors, Supporting Its Infection, Colonization, and Damage to Shrimp

To further investigate the potential pathogenicity of strain VSP1, its biological characteristics were assessed, and whole-genome analysis was performed to elucidate its pathogenic mechanisms. The VSP1 strain exhibited a rapid growth rate, and reached the stationary phase within 18–20 h, indicating that VSP1 is a rapidly growing bacterium that could rapidly proliferate in the shrimp gut once infected, and thus lead to the occurrence of disease [49]. Chromogenic medium is often used for the identification of pathogenic bacteria according to the change in colony color [50]. For example, TCBS agar medium is often used as a preliminary indicator of pathogenicity for *Vibrio*, which showed that the yellow colonies are typically considered non-pathogenic bacteria, and the green colonies are usually identified as pathogens. *V. parahaemolyticus* is one of the most common pathogenic bacteria that form green colonies on TCBS agar medium [51]. Here, VSP1 strain formed smooth, circular green colonies on TCBS agar, indicating its inability to ferment sucrose [52]. This characteristic was further confirmed by Biolog GEN III MicroPlate analysis, which indicated that VSP1 could not use the sucrose as the sole carbon source. Instead, it metabolizes a wide range of other carbon sources, including various monosaccharides, sugar alcohols, amino acids, and organic acids. This is similar with that many pathogenic bacteria prefer to utilize simple compounds, such as monosaccharides, and amino acids, as carbon sources for growth [53].

The virulence of bacteria is closely associated with the genes that involved in the cell motility, adhesion, and quorum sensing (QS) system [16]. The genome of strain VSP1 harbors 57 flagella-related genes, suggesting that it may have considerable motility, which could potentially facilitate its dissemination in the host intestine [54]. This strain also possesses the genes related to mannose-sensitive hemagglutinin (MSHA), toxin-coregulated pilus (TCP), type IV pili, and capsular polysaccharides, which are critical for gut colonization of bacteria by forming the biofilm. Together, genomic features also suggest that VSP1 might have the potential to attach to host tissues, which could be important for initiating and establishing infection [55,56,57,58]. The quorum sensing system plays a crucial role in regulating the expression of virulence genes [59,60]. The genome of VSP1 includes two key autoinducers that could function in parallel to funnel cell-density information internally to control gene expression by QS pathway in *Vibrio* spp. [61], indicating that the virulence of VSP1 might be controlled by the QS system. The Type III Secretion Systems (T3SSs), including two different systems named T3SS1 and T3SS2, play a crucial role in the virulence of Gram-negative pathogens by forming a needle-like structure that transfers effector proteins into host cells, thereby manipulating host cell functions and inducing gastroenteritis [62,63]. T3SS1 is essential for bacterial cytotoxic activity, while T3SS2 is responsible for enterotoxicity [64]. VSP1 contains 34 genes related to T3SS1 and 4 genes associated with T3SS1 secreted effectors, which may contribute to autophagy, cell rounding, and lysis, potentially affecting host cells [65]. By contrast, VSP2 completely lacked T3SS1 and its effectors, suggesting that it may have a reduced ability to directly manipulate host cellular processes. Additionally, VSP1 also harbors the Type II Secretion System (T2SS), which may contribute to bacterial pathogenicity and environmental adaptation by secreting cholera toxin and biofilm matrix proteins (e.g., RbmC, RbmA, Bap1) [66]. This system may not only promote biofilm formation and host colonization but also enhance host-pathogen interactions, thereby amplifying the pathogenic potential of VSP1.

FastANI analysis indicated that VSP1 is clustered together with mode type strain of *V. parahaemolyticus* ATCC 17802, which was reported as the highly lethal pathogenic bacterium of aquatic animals [67]. Since VSP1 causes only enteritis without mortality, its pathogenicity was compared at the genomic level. It is known that *V. parahaemolyticus* could produce three types of hemolysins: thermostable direct hemolysin (TDH), TDH-related hemolysin (TRH), and thermolabile hemolysin (TLH), which are encoded by the *tdh*, *trh*, and *tlh* gene, respectively. Among them, TDH and TRH are considered the most critical virulence factors of *V. parahaemolyticus* [16,68]. *V. parahaemolyticus* ATCC 17802 carries both TDH and TRH (*tdh*+, *trh*+), suggesting high virulence potential. In contrast, VSP1 carries TRH but lacks TDH (*tdh*−, *trh*+). The presence of TRH may contribute to the virulence of VSP1, while the absence of TDH suggests that VSP1 could have relatively mild toxicity. In addition to lacking TDH, VSP1 also lacks all genes associated with T3SS2, a critical virulence factor typically linked to *V. parahaemolyticus* pathogenicity [69,70,71]. Studies have shown that T3SS2 is only found in strains carrying TDH [63,64], suggesting that T3SS2 might cooperate with TDH in the pathogenic process. This further supports the relatively mild virulence of VSP1, as evidenced by our clinical observations, where infected shrimp exhibited rapid enteritis symptoms but did not experience large-scale mortality, either in field outbreaks or under experimental conditions. Moreover, the presence of multiple antibiotic resistance genes suggests that VSP1 represents a potential risk to shrimp farming and environmental health.

## 5. Conclusions

In conclusion, this study identified the specific pathogen of shrimp enteritis, and revealed the potential pathogenic mechanism of this pathogen based on the genome analysis. The typical symptoms of shrimp enteritis were characterized as lethargy, reduced motility, and empty stomach and gut. The taxa belonging to the genus *Vibrio*, particularly OTU1, were significantly enriched in diseased shrimp. Then the corresponding strain VSP1 of OTU1 was directly isolated from the diseased shrimp gut, and its pathogenicity was verified by the fulfillment of Koch’s four postulates. Genomic analysis of VSP1 strain includes 156 putative virulence-associated genes related to adhesion, motility, secretion systems, and toxins, but lacks the TDH toxin and T3SS2 system found in highly pathogenic strains. These results confirm that *V. parahaemolyticus* VSP1 might be the potential pathogen that causes the shrimp enteritis, and provide a basis for early detection and control for shrimp enteritis.

## Figures and Tables

**Table 1:** Genomic characteristics of the isolated strains.

	VSP1	VSP2
**Assembly statistics**		
Genome completeness (%)	100	100
Genome contamination (%)	0.08	2.12
Genome size (bp)	5,366,644	5,603,652
GC content (%)	45.0	45.0
**Prediction and annotation**		
Coding sequences (CDSs)	4829	5037
Average gene length (bp)	320	322
Annotated genes (Bakta)	4276	4471
**Pathogenicity-related genes**		
Genes annotated in VFDB	156	153
Genes annotated in CARD	6	6
Prophage elements	25	17

**Table 2:** The Number of Genes Related to Virulence Factors in *V. parahaemolyticus* ATCC 17802, VSP1 and VSP2.

Virulence Factors	Gene Number		
	*V. parahaemolyticus* ATCC 17802	VSP1	VSP2
**Adherence**			
Mannose-sensitive hemagglutinin (MSHA type IV pilus)	14	15	17
Type IV pilus	4	4	0
Type IV pili (*Yersinia*)	0	1	0
**Antiphagocytosis**			
Capsular polysaccharide	16	15	15
**Motility**			
Flagella	56	57	87
**Iron acquisition system**			
Enterobactin receptors	2	2	0
Heme receptors	2	2	1
Periplasmic binding protein-dependent ABC transport systems	4	4	6
Acinetobactin (*Acinetobacter*)	0	0	5
**Quorum Sensing Factors**			
Autoinducer-2	1	1	1
Cholerae autoinducer-1	1	1	1
**Secretion system**			
EPS type II secretion system	12	12	12
T3SS1	34	34	0
T3SS1 secreted effectors	4	4	0
T3SS2	10	0	0
T3SS2 secreted effectors	1	0	0
AAI/SCI-II T6SS (*Escherichia*)	0	0	2
**Toxin**			
Thermolabile hemolysin	1	1	1
Thermostable direct hemolysin	1	0	0
Alpha-hemolysin (*Escherichia*)	3	0	0
Phytotoxin phaseolotoxin (*Pseudomonas*)	0	0	1
**Endotoxin**			
LOS (*Haemophilus*)	0	1	0
**Acid resistance**			
Urease (*Helicobacter*)	2	0	3
**Glycosylation system**			
O-linked flagellar glycosylation (*Campylobacter*)	1	0	0
**Immune evasion**			
Capsule (*Acinetobacter*)	1	2	0
LPS (*Francisella*)	0	0	1
LPS glucosylation (*Shigella*)	1	0	0
**Others**			
O-antigen (*Yersinia*)	1	0	0

**Table 3:** Antibiotic resistance genes identified in VSP1 and VSP2 strains.

Strain	Predicted ARG	Identity (%)	Drug Class	Resistance Mechanism
VSP1	*CARB-18*	100	β-lactam	Antibiotic inactivation
	*CRP*	95.24	Macrolide, Fluoroquinolone, β-lactam	Antibiotic efflux
	*TxR*	86.29	Tetracycline	Antibiotic efflux
	*parE*	78.98	Fluoroquinolone	Target alteration
	*adeF*	42.68	Fluoroquinolone, Tetracycline	Antibiotic efflux
	*vanT*	33.06	Glycopeptide	Target alteration
VSP2	*CRP*	95.24	Macrolide, Fluoroquinolone, β-lactam	Antibiotic efflux
	*parE*	79.14	Fluoroquinolone	Target alteration
	*tet(B)*	69.59	Tetracycline	Antibiotic efflux
	*PBP3*	45.25	Cephalosporin, Penicillin	Target alteration
	*adeF*	43.15	Fluoroquinolone, Tetracycline	Antibiotic efflux
	*vanT*	33.06	Glycopeptide	Target alteration

**Table 4:** Antibiotic susceptibility profiles of *Vibrio* strains VSP1 and VSP2.

Antibiotic Class	Antibiotics	Concentration (µg)	Diameter of Inhibition Zone (mm)/ Antibiotic Sensitivity	
			VSP1	VSP2
β-lactam	Ampicillin (AMP)	10	0 R	0 R
	Piperacillin (PIP)	100	0 R	0 R
	Cefotaxime (CTX)	30	22 R	22 R
	Cefoxitin (FOX)	30	20 S	20 S
	Cefepime (FEP)	30	19 I	18 R
	Imipenem (IPM)	10	16 R	16 R
	Ceftazidime (CAZ)	30	19 I	19 I
Fluoroquinolones	Levofloxacin (LEV)	5	13 R	15 I
Quinolones	Ciprofloxacin (CIP)	5	15 R	14 R
Tetracyclines	Tetracycline (TE)	30	0 R	12 R
Aminoglycosides	Gentamicin (CEN)	10	12 R	10 R
Chloramphenicols	Chloramphenicol (CMP)	30	32 S	29 S

Note: S, susceptible; I, intermediate; R, resistant.

## Data Availability

The raw sequencing data have been deposited in the NCBI Sequence Read Archive under accession number PRJNA1265796 (https://www.ncbi.nlm.nih.gov/bioproject/PRJNA1265796, accessed on 20 May 2025).

## Supplementary Materials

The following supporting information can be downloaded at: https://www.mdpi.com/article/10.3390/pathogens14111188/s1, Table S1. Genomic information of *Vibrio* type species from the public NCBI database. Table S2. Differences in Virulence Genes between Strain VSP1, VSP2 and *V. parahaemolyticus* ATCC 17802. Figure S1: Alpha diversity of the bacterial community in shrimp gut. Each group consists of 5 biological replicates (*n* = 5).
