# Supplementary material for: Pathogenicity and Genomic Characterization of Vibrio parahaemolyticus VSP1: A Pathogen Linked to Enteritis Outbreak in Shrimp (Penaeus vannamei)
Source: Pathogens. 2025 Nov 20;14(11):1188. doi: 10.3390/pathogens14111188 (PMC12655737; doi:10.3390/pathogens14111188)
Supplement: Supplementary file 1 [file pathogens-14-01188-s001.zip › pathogens-3950687-supplementary.pdf]

Pathogenicity and Genomic Characterization of *Vibrio parahaemolyticus* VSP1: A Pathogen Linked to Enteritis Outbreak in Shrimp (*Penaeus vannamei*)

Jing Wang[1,2], Fengguang Shen[1,2], Meng Tian[1,2], Fanqi Zeng[1,2], Lei Huang[3], Jiayun Yao[3], Can Zong[1,2], Jiong Chen[1,2], Demin Zhang[1,2], Haipeng Guo[1,2]*

[1] State Key Laboratory for Quality and Safety of Agro-Products, School of Marine Sciences, Ningbo University, Ningbo 315211, China

[2] Ministry of Education Key Laboratory of Aquacultural Biotechnology, Ningbo University, Ningbo 315211, China

[3] Zhejiang Institute of Freshwater Fisheries, Huzhou 313001, China

Corresponding author:

*E-mail: guohaipeng@nbu.edu.cn (HPG)

Tel: +86-0574-87600551

Supplementary Table

Table S1. Genomic information of *Vibrio* type species from the public NCBI database.

**Species**	**Strain**	**NCBI Accession number**
Vibrio alginolyticus	ATCC17749	GCF_000354175.2
Vibrio antiquarius	EX25	GCF_000024825.1
Vibrio azureus	LC2-005	GCF_002849855.1
Vibrio campbellii	ATCC25920	GCF_002163755.1
Vibrio harveyi	ATCC14126	GCF_001591145.1
Vibrio hyugaensis	090810a	GCF_002906655.1
Vibrio natriegens	ATCC14048	GCF_001456255.1
Vibrio owensii	XSBZ03	GCF_002021755.1
Vibrio parahaemolyticus	ATCC17802	GCF_001558495.2
Vibrio rotiferianus	B64D1	GCF_002214395.1
Vibrio pelagius	ATCC25916	GCF_024347575.1
Vibrio tubiashii	ATCC19109	GCF_000772105.1
Vibrio fortis	LMG21557	GCF_024347475.1
Vibrio proteolyticus	NBRC13287	GCF_000467125.1
Vibrio chemaguriensis	Iso1	GCF_012275705.1

Table S2.

Differences in Virulence Genes between Strain VSP1, VSP2 and *V. parahaemolyticus* ATCC 17802

Virulence factors	Related genes	V. parahaemolyticus ATCC 17802	VSP 1	VSP 2
Mannose-sensitive hemagglutinin (MSHA type IV pilus)	mshA	2	3	1
	mshB	0	0	1
	mshC	1	1	1
	mshD	1	1	1
	mshE	1	1	1
	mshF	1	1	0
	mshG	1	1	1
	mshH	1	1	1
	mshI	1	1	1
	mshJ	1	1	1
	mshK	1	1	1
	mshL	1	1	1
	mshM	1	1	1
	mshN	1	1	1
	tadA	0	0	1
Type IV pilus	pilA	1	1	0
	pilB	1	1	1
	pilC	1	1	1
	pilD	1	1	1
Type IV pili(Yersinia)	pilW	0	1	0
Capsular polysaccharide	cpsA	1	1	1
	cpsB	1	1	1
	cpsC	1	1	1
	cpsD	1	1	1
	cpsE	1	1	0
	cpsF	1	1	1
	cpsG	1	1	1
	cpsH	1	1	1
	cpsI	1	1	1
	cpsJ	1	1	0
	rmlA	0	0	1
	rmlB	0	0	1
	rmlC	0	0	1
	wbfT	1	0	0
	wbfU	1	0	0
	wbfV/wcvB	1	1	1

	-	0	0	1
	wbfY	1	0	1
	wecA	0	1	1
	wza	1	1	0
	wzb	0	1	0
	wzc	1	1	0
Flagella	cheA	1	1	2
	cheB	1	1	2
	cheR	1	1	1
	cheV	1	1	1
	cheW	1	1	2
	cheY	1	1	2
	cheZ	1	1	2
	filM	1	1	2
	flaA	1	1	1
	flaB	1	1	2
	flaD	2	3	2
	flaE	1	1	1
	flaG	1	1	2
	flaI	1	1	2
	flgA	1	1	1
	flgB	1	1	1
	flgC	1	1	1
	flgD	1	1	1
	flgE	1	1	1
	flgF	1	1	1
	flgG	1	1	1
	flgH	1	1	1
	flgI	1	1	1
	flgJ	1	1	1
	flgK	1	1	1
	flgL	1	1	1
	flgM	1	1	0
	flgN	1	1	1
	flhA	1	1	2
	flhB	1	1	2
	flhF	1	1	2
	flhG	1	1	2
	fliA	1	1	2
	fliD	1	1	2
	fliE	1	1	2
	fliF	1	1	2
	fliG	1	1	2

	**fliH**	**1**	**1**	**2**
	**fliI**	**1**	**1**	**2**
	**fliJ**	**1**	**1**	**2**
	**fliK**	**1**	**1**	**2**
	**fliL**	**1**	**1**	**2**
	**fliN**	**1**	**1**	**2**
	**fliO**	**1**	**1**	**2**
	**fliP**	**1**	**1**	**2**
	**fliQ**	**1**	**1**	**2**
	**fliR**	**1**	**1**	**2**
	**fliS**	**1**	**1**	**2**
	**flrA**	**1**	**1**	**2**
	**flrB**	**1**	**1**	**2**
	**flrC**	**1**	**1**	**2**
	**motA**	**1**	**1**	**1**
	**motB**	**1**	**1**	**1**
	**motX**	**1**	**1**	**1**
	**motY**	**1**	**1**	**1**
**Enterobactin receptors**	**irgA**	**1**	**1**	**0**
	**vctA**	**1**	**1**	**1**
**Heme receptors**	**hutA**	**1**	**1**	**1**
	**hutR**	**1**	**1**	**1**
**Periplasmic binding protein-dependent ABC transport systems**	**vctC**	**1**	**1**	**1**
	**vctD**	**1**	**1**	**1**
	**vctG**	**1**	**1**	**1**
	**vctP**	**1**	**1**	**1**
**Acinetobactin (Acinetobacter)**	**barA**	**0**	**0**	**1**
	**barB**	**0**	**0**	**1**
	**bauB**	**0**	**0**	**1**
	**bauC**	**0**	**0**	**1**
	**bauD**	**0**	**0**	**1**
**Autoinducer-2**	**luxS**	**1**	**1**	**1**
**Cholerae autoinducer-1**	**cqsA**	**1**	**1**	**1**
**EPS type II secretion system**	**epsC**	**1**	**1**	**1**
	**epsE**	**1**	**1**	**1**
	**epsF**	**1**	**1**	**1**
	**epsG**	**1**	**1**	**1**
	**epsH**	**1**	**1**	**1**
	**epsI**	**1**	**1**	**1**
	**epsJ**	**1**	**1**	**1**
	**epsK**	**1**	**1**	**1**
	**epsL**	**1**	**1**	**1**
	**epsM**	**1**	**1**	**1**

	epsN	1	1	1
	gspD	1	1	1
T3SS1 secreted effectors	-	1	1	0
	vopQ	1	1	0
	vopR	1	1	0
	vopS	1	1	0
T3SS1	sycN	1	1	0
	tyeA	1	1	0
	vcrD	1	1	0
	vcrG	1	1	0
	vcrH	1	1	0
	vcrR	1	1	0
	vcrV	1	1	0
	virF	1	1	0
	virG	1	1	0
	vopB	1	1	0
	vopD	1	1	0
	vopN	1	1	0
	vscA	1	1	0
	vscB	1	1	0
	vscC	1	1	0
	vscD	1	1	0
	vscF	1	1	0
	vscG	1	1	0
	vscH	1	1	0
	vscI	1	1	0
	vscJ	1	1	0
	vscK	1	1	0
	vscL	1	1	0
	vscN	1	1	0
	vscO	1	1	0
	vscP	1	1	0
	vscQ	1	1	0
	vscR	1	1	0
	vscS	1	1	0
	vscT	1	1	0
	vscU	1	1	0
	vscX	1	1	0
	vscY	1	1	0
	vxsC	1	1	0
T3SS2 secreted effectors	vopA	1	0	0
T3SS2	-	1	0	0
	-	1	0	0

	-	1	0	0
	-	1	0	0
	-	1	0	0
	-	1	0	0
	-	1	0	0
	vcrD2	1	0	0
	vscC2	1	0	0
	vscN2	1	0	0
**AAI/SCI-II T6SS (Escherichia)**	**aaiI**	**0**	**0**	**2**
**Thermolabile hemolysin**	**tlh**	**1**	**1**	**1**
**Thermostable direct hemolysin**	**tdh**	**1**	**0**	**0**
**Phytotoxin phaseolotoxin (Pseudomonas)**	**cysC1**	**0**	**0**	**1**
**Alpha-hemolysin (Escherichia)**	**hlyA**	**1**	**0**	**0**
	**hlyB**	**1**	**0**	**0**
	**hlyD**	**1**	**0**	**0**
**LOS (Haemophilus)**	**lgtF**	**0**	**1**	**0**
**Urease (Helicobacter)**	**ureB**	**1**	**0**	**1**
	**ureG**	**1**	**0**	**2**
**O-linked flagellar glycosylation(Campylobacter)**	**pseB**	**1**	**0**	**0**
**Capsule(Acinetobacter)**	**-**	**1**	**2**	**0**
**LPS(Francisella)**	**wbtI**	**0**	**0**	**1**
**LPS glucosylation(Shigella)**	**gtrB**	**1**	**0**	**0**
**O-antigen(Yersinia)**	**cpsB**	**1**	**0**	**0**

Supplementary Figure

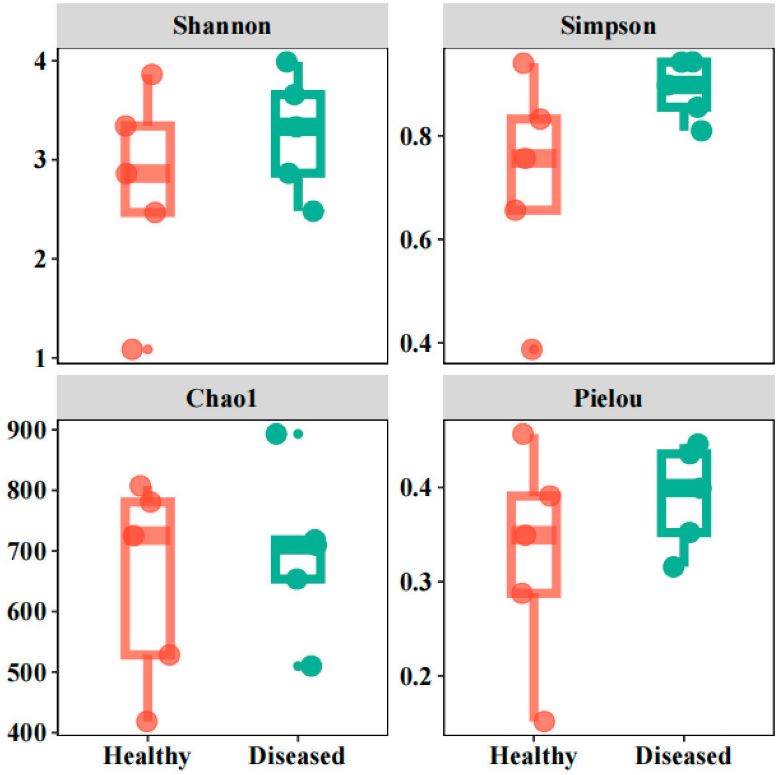

Figure S1. Alpha diversity of the bacterial community in shrimp gut. Each group consists of 5 biological replicates (n = 5).
